# Klinefelter Syndrome Presenting as Suicidal Attempt

**DOI:** 10.7759/cureus.10667

**Published:** 2020-09-26

**Authors:** Mandeep Singla, Abhinav Gupta

**Affiliations:** 1 General Medicine, Government Medical College and Hospital, Chandigarh, IND; 2 General Medicine, Government Medical College and Hospital, Chandigarh, IND

**Keywords:** klinefelter syndrome, suicide, depression

## Abstract

Klinefelter syndrome is the most common sex chromosome disorder, manifested as hypogonadism, gynecomastia, and impaired spermatogenesis. It is characterized by the presence of one or more extra X chromosomes. Patients with Klinefelter syndrome are highly susceptible to psychiatric disturbances as compared to the general population. These include personality changes, depression, and psychosis. Rarely, these psychiatric disturbances can be the presenting manifestations in these individuals. Hence, meticulous clinical examination should be performed in every patient of psychiatric illness to rule out systemic illness like hypogonadism, as the treatment of underlying medical condition can be beneficial in alleviation of the psychiatric illness.

## Introduction

Klinefelter syndrome with the classic 47, XXY karyotype is a common chromosomal abnormality affecting one in 500-1,000 males. It was first described in 1942 [1]. Onset of puberty is normal, but testicular failure supervenes, resulting in small firm testes, azoospermia, and a variable degree of androgen deficiency. The classical description of Klinefelter syndrome is a tall male, with narrow shoulders, broad hips, sparse body hair, gynecomastia, small testes, androgen deficiency, and reduced intelligence [2]. Due to variable androgen deficiency, about 70% remain undiagnosed throughout the lifespan [3]. We report a patient who presented with suicidal poisoning and was subsequently diagnosed as Klinefelter syndrome.

## Case presentation

**Figure 1:**
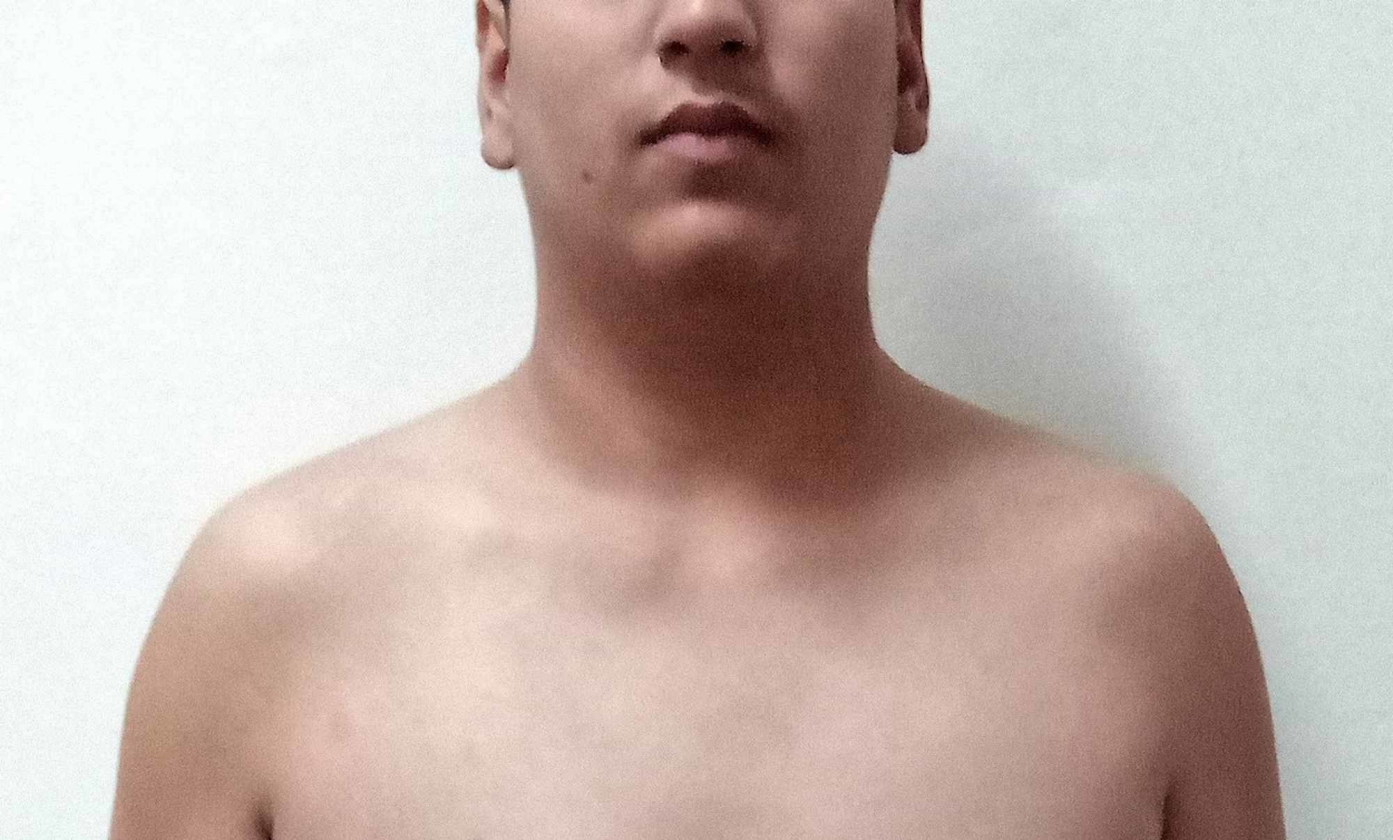
Absent beard and moustache at the age of 20 years in the index case

A 20-year-old male presented to the emergency department with vomiting, excessive retching, and diarrhea after consuming organophosphate insecticide in a suicidal attempt. General physical examination revealed miosis, hyperhidrosis, and hypersalivation. On chest auscultation, bilateral crepitations were present. Gastric lavage was performed immediately. Complete atropinization was done followed by maintenance infusion of atropine for two days which was gradually tapered off. Pralidoxime was also given for 24 hours. The patient responded to the treatment. However, it was noticed that he is not having beard and moustache despite being 20 years of age (Figure 1).

**Figure 2:**
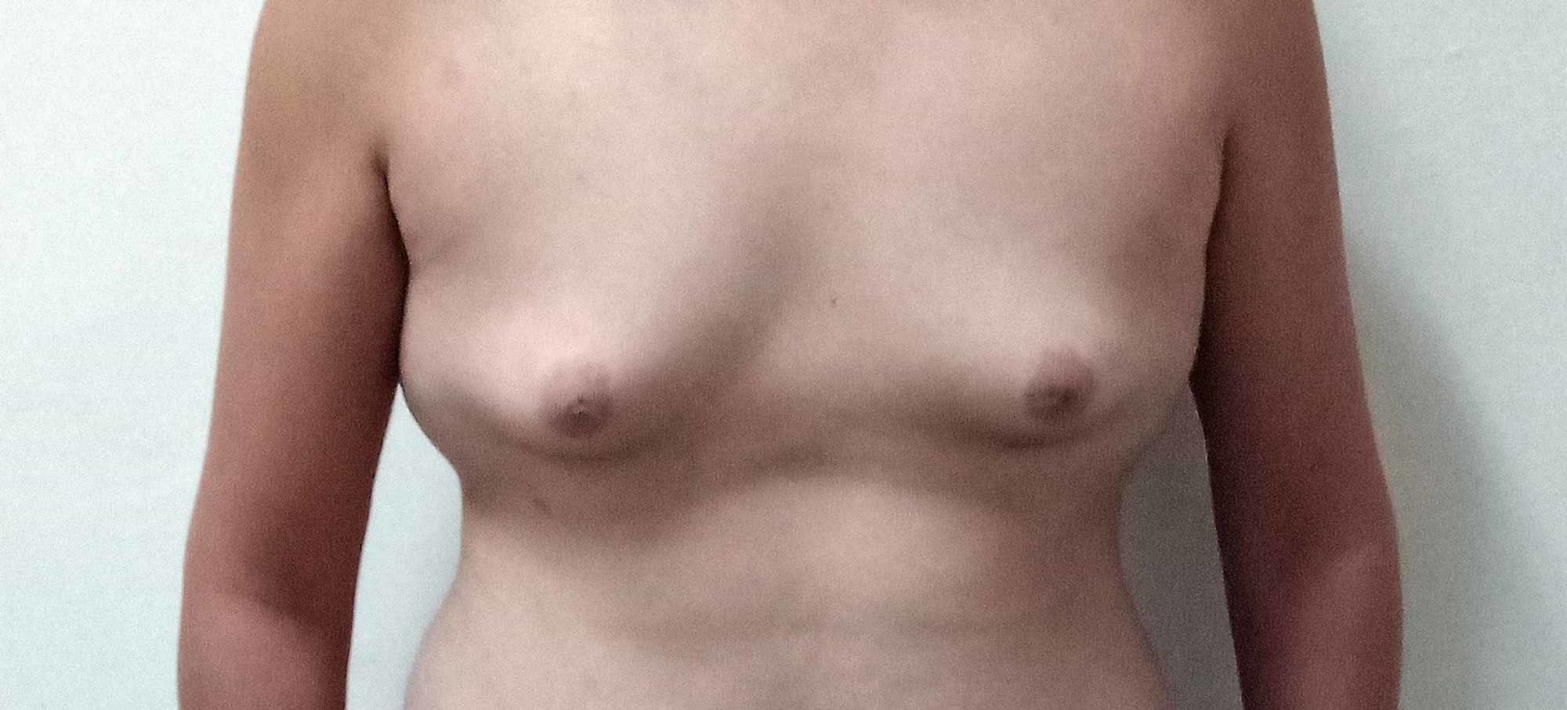
Bilateral gynecomastia and absent body hair in the index case

He expressed feeling of insecurity about physique, learning difficulties, and perceived rejection from peers. On further evaluation, he was tall with a height of 178 cm, weight 78 kg, and a BMI of 24.6 kg/m^2^. Axillary and chest hair were absent. Testes were firm in consistency with a volume of 2 ml bilaterally .Gynecomastia was present (Figure 2).

Hormonal profile revealed high follicle-stimulating hormone (FSH) 45 mIU/ml (normal range = 1.5-12.4 mIU/ml), high luteinizing hormone (LH) 12.8 mIU/ml (normal range = 1.7-8.6 mIU/ml), and low testosterone 1.4 nmol/L (normal range = 9.9-27.8 nmol/L), suggestive of hypergonadotropic hypogonadism. Karyotype of peripheral blood mononuclear cells revealed 47, XXY karyotype, thus confirming the diagnosis of Klinefelter syndrome. The patient was started on testosterone replacement therapy, along with counseling and psychotherapy.

## Discussion

Patients with Klinefelter syndrome are highly prone to psychiatric disturbances as compared to the general population, including personality changes, neurotic disorders, and psychosis. Personality disorders are the most frequently reported psychiatric manifestations. They have features of passivity, dependency, low social drive, self-concern, limited interest and skills, lability, manipulativeness, and suspiciousness. They have difficulty in coping with challenges and frustration [4]. In addition, there is a significant level of ignorance that prevents parents from seeking early medical opinion for these individuals, especially in developing countries. Among neurotic disorders, there is predominance of hysterical features, conversion reaction, hypochondriasis, and reactive depression. However, these often go unnoticed due to neurotic compensatory mechanisms [5]. Depression has been attributed to the state of hypogonadism, which results in decreased sexual drive leading to loss of libido. The premature loss of libido results in feeling of inadequacy, and consequently depression and suicidal ideation [6]. Learning disabilities and presence of gynecomastia further aggravates the condition. These individuals often do not seek medical attention due to perceived social stigma associated with the state of hypogonadism. Depression has been shown to respond to testosterone replacement therapy in these individuals. The index case expressed feeling of insecurity about physique, learning difficulties, and perceived rejection from peers. However, he did not seek medical advice previously due to social inhibition and landed up in hospital with suicidal attempt. Thus, meticulous clinical examination should be performed in every patient of psychiatric illness to rule out systemic illness like hypogonadism. Furthermore, treatment of the underlying medical condition can also be beneficial in alleviation of the psychiatric illness.

## Conclusions

Psychiatric manifestations in patients with Klinefelter syndrome include psychosis, neurotic disorders, and personality changes. Rarely, these psychiatric disturbances can be the presenting manifestations in these individuals. Depression has been attributed to the state of hypogonadism, which results in loss of libido and consequently, feeling of inadequacy, depression, and suicidal ideation. Depression has been shown to respond to testosterone replacement therapy in these individuals.
